# Methods of investigation transformation kinetics of yttrium carbonate hydroxide in citric acid solution into yttrium citrate dihydrate

**DOI:** 10.1016/j.mex.2020.101153

**Published:** 2020-11-27

**Authors:** W. Janusz, E. Skwarek, D. Sternik, S. Pikus, D. Pawlak, J.L. Parus, R. Mikołajczak

**Affiliations:** a Institute of Chemical Sciences, Faculty of Chemistry, Maria Curie-Sklodowska University, Maria Curie-Sklodowska Sq. 3, Lublin 20 031, Poland; b Radioisotope Centre Polatom, National Centre for Nuclear Research, Andrzej Soltan str. 7, Otwock 05 400, Poland

**Keywords:** Yttrium citrate dihydrate, Synthesis of radiopharmaceuticals, Kinetics transformation

## Abstract

A method of synthesis crystalline yttrium citrate dihydrate was proposed as a result of the transformation of the freshly precipitated basic yttrium carbonate phase in a citric acid solution. The synthesis time was determined on the basis of composition analysis, structure and thermogravimetric studies of samples taken during the synthesis. The research methods used have shown that in the initial stage of the synthesis, the processes of citric acid sorption on basic yttrium carbonate and transformation of amorphous yttrium carbonate hydroxide into crystalline yttrium hydroxide occurs. It is only after 72 h of synthesis that the crystalline yttrium citrate dihydrate is formed.

• Synthesis crystalline yttrium citrate dehydrate.
• The synthesis time 72 h.
• Synthesis components: the freshly precipitated basic yttrium carbonate phase in a citric acid solution.

## Graphical abstract

**Figure:**
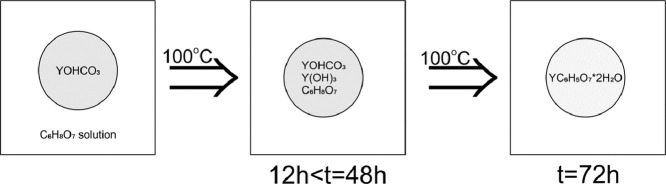

Specifications table

**Table:** 

Subject Area:	Chemistry
More specific subject area:	*Yttrium citrate synthesis*
Method name:	Physicochemical characterization of Transformation Kinetics of Yttrium Carbonate Hydroxide in Citric Acid Solution into Yttrium Citrate Dihydrate
Name and reference of original method	**Original references:** R.S. Zhou, J.F. Song, Q.F. Yang, X.Y. Xu, Q. J. Xu, T.G. Wang, Syntheses, structures and magnetic properties of a series of 2D and 3D lanthanide complexes constructed by citric ligand, J. Molecular Struct. 877 (2008): 115–122. R. Baggio and M. Perec, Isolation and Characterization of a Polymeric Lanthanum Citrate. Inorg. Chem. 43(2004): 6965–6968.
Resource availability:	**Abberviations** CH analysis, analysis of the H and C contents PXRD – powder X- ray diffraction analysis TG- thermograwimetric analysis Rwp-weighted residual-Maud software Sig-goodness of fit Maud software

## Introduction

The synthesis and properties of rare-earth elements citrates are studied not only from the theoretical point of view but also because of their practical application in modern technologies and yttrium citrate belongs to such relationship compound. Metal citrates were synthesized by precipitation reactions from homogeneous solutions of soluble metal salts and citric acid or sodium citrate [1], [2], [3]. Another method for obtaining the dispersion of metal citrates is the phase transformation method involving the reaction of a metal hydroxide or oxide with citric acid [4], [5], [6]. This method belongs to the group of methods that allow obtaining monodisperse colloids [7]. In the mentioned papers of transformation of metal hydroxide in a solution of citric acid into metal citrate, the structure, composition, thermal and spectroscopic properties of the obtained product were analyzed.

This paper contains supplementary information about kinetic of synthesis of monoclinic yttrium citrate dihydrate by phase transformation of amorphous yttrium carbonate hydroxide in citric acid solution [8]. The synthesis time was determined on the basis of composition (CH analysis), structure (PXRD method) and thermogravimetric studies of samples taken during the synthesis. The research methods used have shown that in the initial stage citric acid sorption on yttrium carbonate hydroxide and transformation of carbonate into crystalline yttrium hydroxide occurs. It is only after 72 h of synthesis that the crystalline yttrium citrate dihydrate is formed.

## Materials and methods

Reagents and equipment
Yttrium chloride (YCl_3_⋅6 H_2_O) Sigma - Aldrich
Urea (CH_4_N_2_O) of the Polish Chemical Reagents
Citric acid (C_6_H_8_O_7_ * H_2_O) of the Polish Chemical Reagents
Autoclave Engineers reactor
Perkin - Elmer CHN analyzer
Derivatograph-C apparatus (F. Paulik, J. Paulik, L. Erdey)
HZG – 4 Carl Zeiss Jena X-ray powder diffractometer

## Procedure

Yttrium citrate dihydrate was obtained by transformation of freshly precipitated yttrium carbonate hydroxide in a citric acid solution.

In order to obtain yttrium carbonate hydroxide, the procedure given by Sprycha and Matijevic [9] was used, i.e.: solutions of: 0.02 mol/dm^3^ yttrium chloride solution, 0.2 mol/dm^3^ urea solution were prepared, which were then filtered through 0.22 µm Milipore filters. Then 400 cm^3^ of 0.02 mol/dm^3^ yttrium chloride solution was mixed with 400 cm^3^ of urea solution and the mixture was heated in a 1000 cm^3^ beaker in a heating basket. After the appearance of a white precipitate, the suspension was continued to be heated for one hour before it was cooled in an ice-water bath. The resulting precipitate of basic yttrium carbonate (Y(OH)CO_3_) was filtered on a Millipore membrane filter with a pore size of 0.22 µm. The filtered precipitate was ultrasonically dispersed in redistilled water, and the suspension was then filtered. The purification procedure was repeated until the filtrate conductivity was constant.

In order to obtain yttrium citrate dihydrate, washed yttrium carbonate hydroxide obtained from two syntheses (about 2 g) was placed in 200 cm^3^ of 0.1 mol/dm^3^ citric acid solution [8]. The suspension was then sonicated for 3 min, the pH was measured (pH = 2.71) and then transferred to an Autoclave Engineers reactor and heated at 100 °C for 3 days while stirring at 700 rpm. During the synthesis, after 12, 24, 48 and 72 h, samples of the slurry were taken from the reactor, filtered on a 0.22 µm Millipore filter, the filter cake washed and then dried at room temperature. The dried sediment was analyzed for the content of C and H as well as thermogravimetric and PXRD tests.

## Results and disicussion

The transformation time of yttrium carbonate hydroxide in a solution of citric acid into yttrium citrate was determined on the basis of results C and H analysis and thermogravimetric and PXRD studies of samples taken during the synthesis (after 12, 24, 48 and 72 h).

Using the CHN analyzer the following results of the percentage of the elements: carbon (C) and hydrogen (H) in the samples prepared as a result of hydrothermal synthesis at 100 °C were obtained, Table 1. The analysis of the samples no 1–4 composition for C and H contents indicates that during 48 h of synthesis the composition of the samples is close to that of citric acid (37.5% C and 4.2% H). This indicates that the analyzed samples contain a significant amount of unreacted citric acid that may have been absorbed on the tested sediment, this amount was calculated taking into account: the contents of C (3 column in Table 2), calculated content of carbon in yttrium hydroxide carbonate (6.63%) and calculated content of carbon in citric acid (37, 5%), the results of the calculations are shown in column 7 of Table 1. Analysis of C and H composition of sample taken after 72 hour of synthesis indicate that after this time of synthesis the yttrium citrate dihydrate was formed [8].

**Table 1:** The results of CHN analysis – the sediment samples taken during the yttrium citrate synthesis at temperature 100 °C.

Sample	Synthesis time [h]	Contents of C and H in the sample		Calculated contents of C and H in YCit*2H_2_O		%Contents of citric acid
		C [%]	H [%]	C [%]	H [%]	[%]
YOHCO_3_	0	6.8	1.6	22.95	2.89	
1.	6	36.3	4.2			96.2
2.	12	36.7	4.2			97.3
3.	24	36.4	4.2			96.5
4.	48	36.7	4.2			97.5
5.	72	25.4	3.0

**Table 2:** Results of Rietveld's refinement using Maud software,.

Sample no	Compound	A [Å]	B [Å]	C [Å]	Beta [deg]	Sig	Rwp [%]	H_3_Cit [%]
2	H_3_Cit	12.8115	5.6131	11.4665	111.25	0.36	13.77	95.4
	Y(OH)_3_	6.261	6.261	3.544				
3	H_3_Cit	12.8135	5.6183	11.4703	111.24	0.43	12.59	96.9
	Y(OH)_3_	6.261	6.261	3.544				
4	H_3_Cit	12.8059	5.6172	11.4652	111.27	0.47	13.94	96.5
	Y(OH)_3_	6.261	6.261	3.544				

The use of freshly prepared yttrium carbonate hydroxide as starting reagent in the synthesis of yttrium oxide results lower temperature synthesis in comparison to conditions of synthesis of other metal citrates [4], [5].

**Fig. 1:**
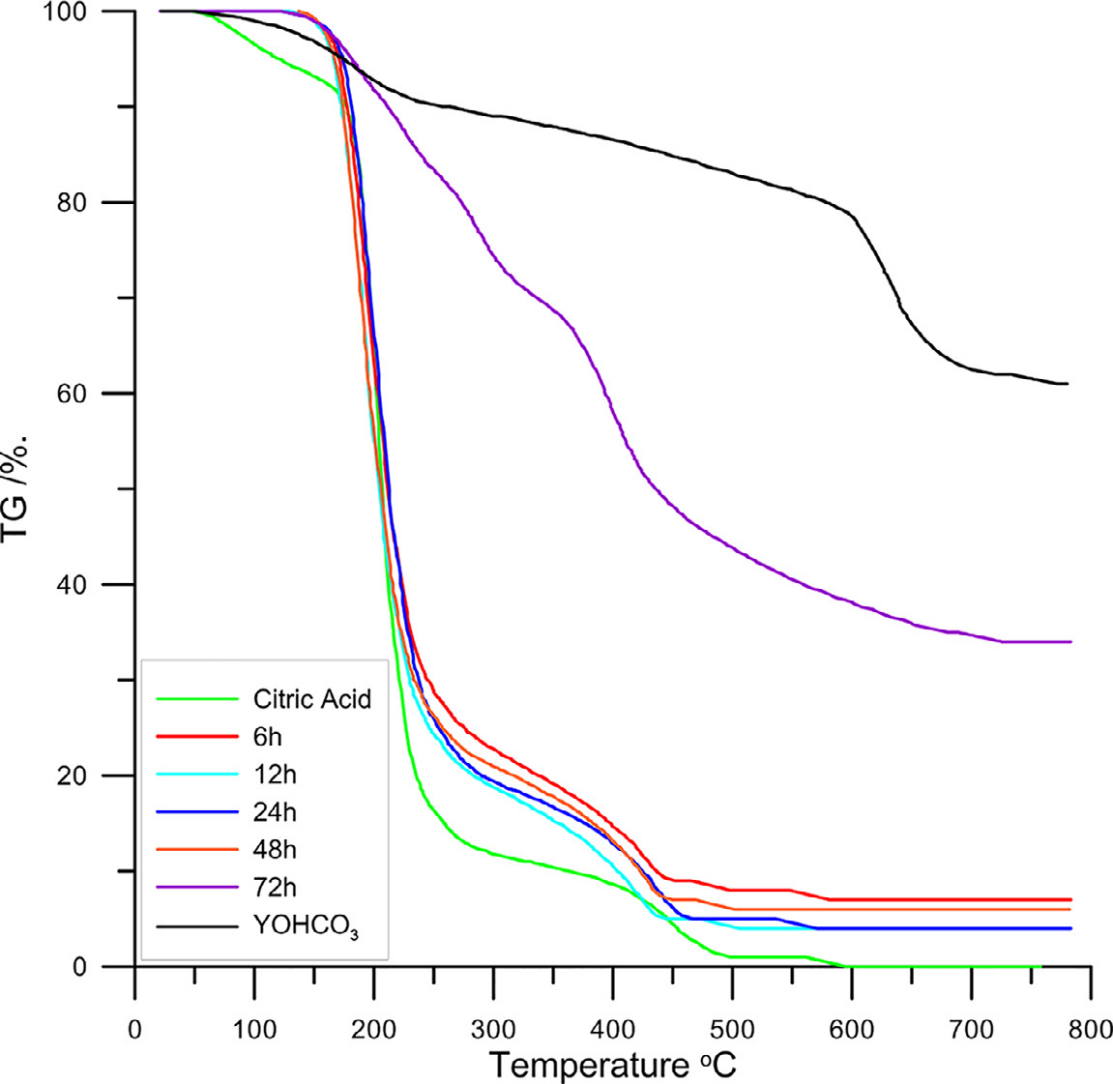
TGA curves of the citric acid sample, yttrium carbonate hydroxide samples and the samples taken after the 6, 12, 24, 48 and 72 h-synthesis.

The course of the gravimetric analysis curves presented in Fig. 1 and indicates that the thermal decomposition of the tested compounds is multistage. Additionally for comparison the course of thermogravimetric curves for citric acid and yttrium carbonate hydroxide as the reference systems (starting reagents) is presented in this Fig. 1. Up to a temperature of 150 °C, the TG curve of citric acid is characterized by a slight (several percent) weight loss associated with the loss of hygroscopic water [10]. Above this temperature up to 300 °C there is a significant weight loss (~ 81%) associated with the decomposition of citric acid in the synthetic air [11]. The further weight loss associated with the oxidation of citric acid occurs up to 550 °C which is visible on the TG curves for the samples taken after 6, 12, 24 and 48 h. The thermogravimetry curves of the synthesized materials from 150 to 200 °C coincide with the TG curve of citric acid. The largest weight loss (~ 76%) for these samples is observed up to 258 °C. After heating the samples taken above 600 °C, 7, 4, 4, and 6% of the initial mass remains, respectively. This indicates a low content of yttrium compounds in the analyzed samples. Considering the analysis for C and H contents, it can be assumed that the samples contained a significant amount of unreacted citric acid. The course of TG curve of the sample taken after 72 h of synthesis differ significantly from previous one and percent of remains of mass after heating it above 700 °C agree with contents of yttrium oxide in yttrium citrate dihydrate [8].

**Fig. 2:**
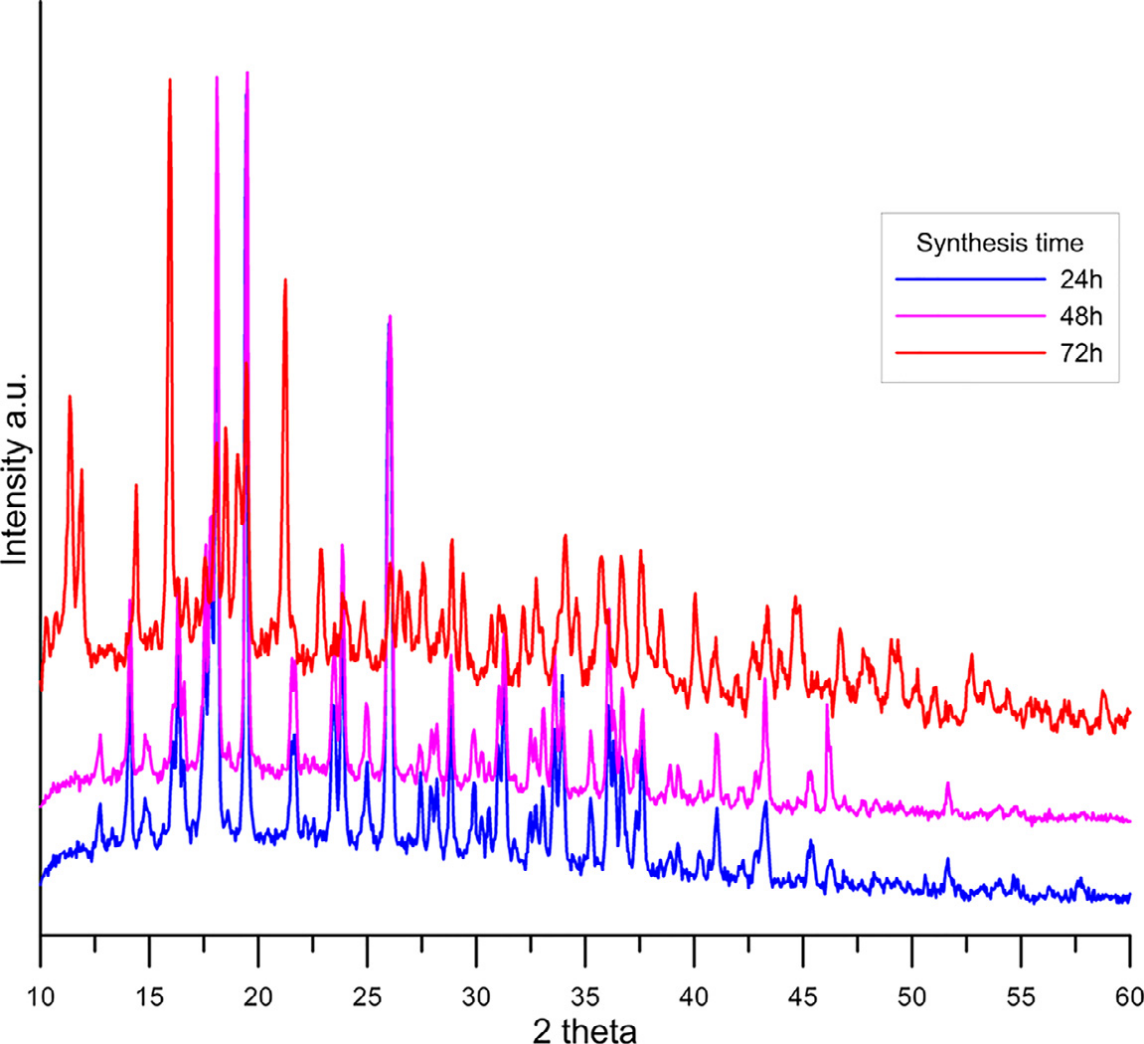
PXRD pattern of the samples taken after 24, 48 and 72 h of synthesis.

The samples taken from the reactor after 12, 24, 48 and 72 h synthesized at 100 °C were subjected to the XRD analysis. The diffraction patterns of the samples (except for the sample after 12 h) are given in Fig. 2. The analysis of the reflection in the PXRD pattern of the samples taken during the synthesis after 12, 24 and 48 h of synthesis indicated the presence of reflections characteristic of the citric acid phase and smaller peaks that can be attributed to yttrium hydroxide. However, no reflections characteristic of yttrium carbonates were found (the diffraction pattern were analyzed for the presence of yttrium hydroxycarbonate, yttrium oxycarbonate and yttrium carbonate). Next the PXRD pattern of these samples was refined by means of the Rietveld method using the Maud software [12]. As the initial models for the refinement the CIF files was used [13,14]. The calculated lattice constants using the Maud software, the fitting parameters (sig and Rwp) and contents of citric acid are collected in Table 2.

**Fig. 3:**
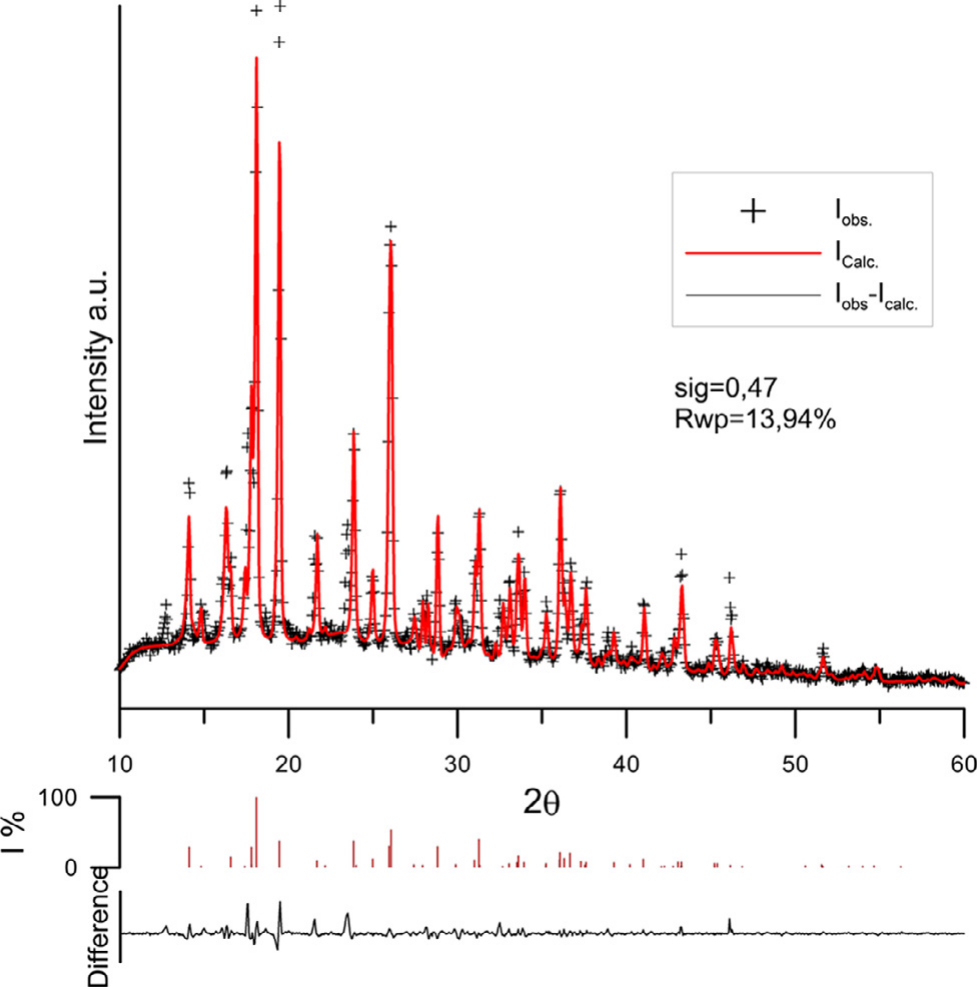
XRD pattern of sample taken after 48hours of synthesis after Rietveld's refinement using Maud software. Symbols represents experimental points, red line fit of Rietveld method, and black line difference between experimental and calculated results.

An example of fitting of calculations to the diffraction pattern for sample no 2 is shown in Fig. 3. The phase analysis carried out confirms the results obtained with the TG method with a high content of citric acid in the samples taken during synthesis. It should be noted that the amount of amorphous phase in the sample, which could have been yttrium carbonate hydroxide, was not determined by XRD. In turn, calculations based on the carbon content of samples no 2 −4 did not take into account the presence of yttrium hydroxide, which could have arisen as a result of hydrolysis of yttrium hydroxide carbonate during the synthesis of yttrium citrate The results of the content of yttrium carbonate hydroxide and yttrium hydroxide are given in Table 3. As can be seen, the content of both compounds in the tested samples is comparable. The total content of yttrium forms in the sample can be determined on the basis of TG analysis by converting the content of yttrium oxide after heating the sample to 750 °C based on the distribution of equal amounts of yttrium hydroxide carbonate and yttrium hydroxide. A summary of the results of the content of yttrium forms in the samples taken during the synthesis is given in Table 4. As can be seen, the sum of the calculated content of yttrium hydroxide carbonate (column 1 of Table 4) based on the content of carbon and yttrium hydroxide determined by the XRD method is close to that estimated on the basis of thermogravimetric analysis of carbonate content (last column of the Table 3).

**Table 3:** Calculated amount of yttrium species in samples collected during the synthesis of yttrium citrate.

Sample no	YOHCO_3_ Y(OH)_3_ [%]	YOHCO3 [%]	Y(OH)3 [%]	Sum of column 3 + 4 [%]
1	9.41	3.8		
2	8.07	2.7	4.6	7.3
3	5.38	3.5	3.0	6.5
4	8.07	2.5	3.5	6.0

## References

[1] Petrova N., Todorovsky D. (2006). Thermal decomposition of zirconium–yttrium citric complexes prepared in ethylene glycol and water media. Mater. Res. Bull..

[2] Babeshkina Z.M., Martynenko L.I. (1966). About yttrium citrate (Russian text). Zh. Neorg. Khim..

[3] Babeshkina Z M., Martynenko L.I., Grigor'ev A.I. (1966). About hydroxy rare earth elements citrate(Russian text). Zh. Neorg. Khim..

[4] Zhou R.S., Song J.F., Yang Q.F., Xu X.Y., Xu Q J., Wang T.G. (2008). Syntheses, structures and magnetic properties of a series of 2D and 3D lanthanide complexes constructed by citric ligand. J. Mol. Struct.

[5] Liu S.G., Liu W., Zuo J.L., Li Y.Z., You X.Z. (2005). Synthesis, structure and luminescent properties of lanthanide(III) polymeric complexes constructed by citric acid. Inorg. Chem. Commun..

[6] Baggio R., Perec M. (2004). Isolation and characterization of a polymeric lanthanum citrate. Inorg. Chem..

[7] Matijevic E. (1986). Monodispersed colloids: art and science. Langmuir.

[8] Janusz W., Skwarek E., Sternik D., Pikus S., Pawlak D., Parus J.L., Mikołajczak R. (2020). Synthesis of yttrium citrate from yttrium carbonate hydroxide and citric acid. Materials Chemistry and Physics.

[9] Sprycha R., Jabłoński J., Matijevic E. (1992). Zeta potential and surface charge of monodispersed colloidal yttrium (iii) oxide and basic carbonate. J. Colloid Interface Sci..

[10] Peng C., Chow A.H.L., Chan C.K. (2001). Hygroscopic study of glucose, citric acid, and sorbitol using an electrodynamic balance: comparison with UNIFAC predictions. Aerosol. Sci. Tech..

[11] Wiecinska P. (2016). Thermal degradation of organic additives used in colloidal shaping of ceramics investigated by the coupled DTA/TG/MS analysis. J. Therm. Anal. Calorim..

[12] Ferrari M., Lutterotti L. (1994). Method for the simultaneous determination of anisotropic residual stresses and texture by X-ray diffraction. J. Appl. Phys..

[13] Glusker J.P., Minkin J.A., Patterson A.L. (1969). X-ray crystal analysis of the substrates of aconitase. IX. A refinement of the structure of anhydrous citric acid. Acta Crystallogr. Sec. B.

[14] Beall G.W., Milligan W.O., Wolcott H.A. (1977). Structural trends in the lanthanide trihydroxides. J. Inorg. Nucl. Chem..

